# High Expression of ATP6V1C2 Predicts Unfavorable Overall Survival in Patients With Colon Adenocarcinoma

**DOI:** 10.3389/fgene.2022.930876

**Published:** 2022-09-21

**Authors:** Guanghua Li, Jiahua Huang, Sile Chen, Yulong He, Zhixiong Wang, Jianjun Peng

**Affiliations:** ^1^ Gastrointestinal Surgery Center, Sun Yat-sen University First Affiliated Hospital, Guangzhou, China; ^2^ Digestive Medical Center, Sun Yat-sen University Seventh Affiliated Hospital, Shenzhen, China

**Keywords:** colon adenocarcinoma, ATP6V1C2, prognosis, metastasis, EMT, Wnt signaling pathway

## Abstract

**Aims:** Colon adenocarcinoma (COAD) is responsible for 90% of all colorectal cancer cases and is one of the most common causes of cancer-related deaths worldwide. ATP6V1s (cytosolic V1 domain of vacuolar adenosine triphosphatase) participate in the biological process of transporting hydrogen ions and are implicated in tumor growth and metastasis. ATP6V1C2 as a family member has been documented to associate with esophageal carcinoma and renal clear cell carcinoma, while its roles in COAD remain elusive.

**Methods:** The expression status, potential molecular mechanism, and prognostic value of ATP6V1C2 in COAD were investigated using The Cancer Genome Atlas (TCGA) and Gene Expression Omnibus (GEO) databases. In addition, its biological roles in COAD were explored through *in vitro* studies.

**Results:** ATP6V1C2 showed a significantly higher expression level in COAD compared with matched non-cancerous tissues. High expression of ATP6V1C2 predicted a shorter overall survival both in TCGA and GEO COAD datasets, and ATP6V1C2 was identified as an independent factor associated with overall survival in COAD. Bioinformatic analyses showed that high expression of ATP6V1C2 was associated with high epithelial–mesenchymal transition (EMT) score and Wnt signaling pathway was significantly enriched from differentially expressed genes between ATP6V1C2-high and -low group. We also found that high expression of ATP6V1C2 could decrease pathway activity of CD8 T effector implicated in tumor microenvironment (TME). *In vitro* study revealed that ATP6V1C2 knockdown resulted in aberrant expression of Wnt- and EMT-related genes and inhibited COAD cell proliferation and growth.

**Conclusion:** This is the first study to reveal the molecular functions of ATP6V1C2 in COAD. Our study suggests that overexpressed ATP6V1C2 might promote EMT by activating Wnt signaling pathway, resulting in cancer metastasis and poor prognosis. This study paves the way for understanding potential molecular mechanisms and therapeutic perspectives in COAD.

## Introduction

Colorectal cancer (CRC) is one of the most aggressive gastrointestinal cancers worldwide, which accounts for 8% of all cancer-related deaths (Ferlay et al., 2019). Colon adenocarcinoma (COAD) is the most common histological subtype, which is responsible for 90% of all CRC cases (Barresi et al., 2015). In China, CRC is the fifth most common cancer and the fifth most common cause of cancer death (Chen et al., 2016). Despite recent advances in therapy for COAD, such as chemotherapy, radiotherapy, and immunotherapy, the prognosis of COAD patients remains poor, especially when metastases to the lymph nodes or distant organs are present. Further studies are therefore needed to clarify the molecular pathogenesis of COAD to explore new therapeutic possibilities.

Vacuolar adenosine triphosphatase (V-ATPase), a multisubunit enzyme, is widely distributed in eukaryotic cells and transports H+ by hydrolyzing ATP that mediates acidification of eukaryotic intracellular organelles (Couto-Vieira et al., 2020; Santos-Pereira et al., 2021). V-ATPase is composed of a cytosolic V1 domain (also called ATP6V1 containing the ATP catalytic site) and a transmembrane V0 domain. The V1 domain consists of three A, three B, and two G subunits, as well as a C, D, E, F, and H subunits. Previous studies have shown that ATP6V1 plays vital roles in various disease conditions, such as distal renal tubular acidosis (Cornière and Eladari, 2020; Jobst-Schwan et al., 2020), tumors including renal clear cell carcinoma (Li et al., 2020) and esophageal carcinoma (Couto-Vieira et al., 2020), abnormal bone development, and diabetes (Hayek et al., 2019; Santos-Pereira et al., 2021). However, the prognostic roles of ATP6V1C2 in colon cancer have not been documented so far.

In the present study, we investigated the association between the ATP6V1C2 expression and overall survival in COAD patients and the potential molecular mechanism of ATP6V1C2 involved in COAD. This study could pave the way for understanding potential molecular mechanisms and diagnostic/therapeutic perspectives in COAD.

## Materials and Methods

### Datasets

The transcriptome data (level 3) of RNA-seq of COAD and matched non-cancerous tissues (NC) were obtained from The Cancer Genome Atlas (TCGA, https://portal.gdc.cancer.gov/). The RNA-seq transcriptome data and microsatellite instability (MSI) MANTIS score of patients were downloaded from TCGA and cBioPortal (http://www.cbioportal.org/), respectively. We also retrieved the clinical data, including demographic information, survival endpoint (vital status, days to death, and day to last follow-up), stage and histological subtype, and MSI status. The raw read counts were utilized to identify differentially expressed genes (DEGs) between COAD and NC by R package DEseq2 (https://www.bioconductor.org/packages/release/bioc/html/DESeq2.html). We calculated fragments per kilobase of exon model per million mapped fragments (FPKM) of each gene in normal and tumor samples in TCGA COAD. The up-regulated or down-regulated gene was defined with a false discovery rate (FDR) value <0.05, the log_2_ (fold change [FC]) > 1 or < −1, respectively.

Gene expression microarray data and clinical information of GSE29623 (Chen et al., 2012) and GSE71187 (An et al., 2015) datasets were downloaded from GEO datasets. Probe IDs were matched to gene symbols using the GPL570 platform (Affymetrix Human Genome U133 Plus 2.0 Array) and GPL6480 (Agilent-014850 Whole Human Genome Microarray 4 × 44 K G4112F), respectively. The mean expression value of the probes is used as the expression value for the gene in question if multiple probes were mapped to a single gene. For GSE29623 dataset, mean expression of two probes (1552532_a_at and 1553989_a_at) were calculated for each gene. In GSE71187 dataset, the expression level of probe of A_23_P250914 was calculated for each gene. The expression level of each gene of GSE29623 and GSE71187 was evaluated by the expression of probes. Patients with unknown survival status and survival time were excluded from the further analyses.

### Pathway Enrichment Analysis

Pathway analyses were based on DEGs between COAD and NC or between ATP6V1C2-high and -low groups, which were performed in the Gene Ontology and/or Kyoto Encyclopedia of Genes and Genomes (KEGG) using the all canonical pathways as NCBI Gene IDs (c2.cp.v7.4.entrez.gmt). KEGG pathways with normalized enrichment score (NES) > 2 and FDR <0.05 were selected for further analysis.

### Gene Set Variation Analysis on CD8 T Effector Pathways

The gene sets representing CD8^+^ T effector pathway were obtained from the previous study (Mariathasan et al., 2018). CD8 T effector pathway encompasses nine genes, including *CD8*, *CD4*, *CXCL10*, *CXCL9*, *GZMA*, *GZMB*, *IFNG*, *PRF1*, and *TBX21* (Rosenberg et al., 2016; Mariathasan et al., 2018). The enrichment scores of the gene set in each COAD sample were quantified by ssGSEA algorithm using R package gene set variation analysis (GSVA) (Hänzelmann et al., 2013).

### Epithelial–Mesenchymal Transition Score Analysis

A 76-gene epithelial–mesenchymal transition (EMT) signature (76GS) was developed and validated using gene expression from non-small cell lung cancer (NSCLC) cell lines, and patients treated in the BATTLE trial were applied in the present study. EMT score based on the 76GS method was calculated as previously described (Byers et al., 2013) for each tumor tissue sample derived from TCGA COAD cohort.

### Gene Set Enrichment Analysis

In this study, the gene sets of all canonical pathways as NCBI Gene IDs (c2.cp.v7.4.symbols.gmt) from MsigDB database (http://www.gsea-msigdb.org/gsea/msigdb/index.jsp) were used for Gene Set Enrichment Analysis (GSEA) was performed with R package clusterProfiler. NES and FDR were both calculated. The significant pathway was defined as NES ≥2 and FDR <0.05. An array of classical pathways involved in tumorigenesis is included in gene set cancer analysis (GSCA,//bioinfo.life.hust.edu.cn/GSCA/#/), including apoptosis, cell cycle, DNA damage response, EMT, hormone AR, hormone ER, PI3K/AKT, RAS/MAPK, RTK, and TSC/mTOR pathways. The crosstalk between DEGs related to Wnt signaling pathway and EMT process was investigated based on GSCA.

### Protein–Protein Interaction Network

In this work, GeneMANIA (http://genemania.org) and STRING (https://string-db.org/cgi/input.pl) databases as online tools were used to predict co-expressed genes with ATP6V1C2. Top 20 genes that interacted with ATP6V1C2 retrieved from GeneMANIA were used for protein–protein interaction network construction. The protein–protein interaction network between DEGs in COAD vs. NC among these top 20 genes and ATP6V1C2 was constructed according to STRING.

### Cell Lines, Small Interfering RNAs, and Cell Transfection

Two human colon cancer cells SW480 and HCT116, purchased from National Collection of Authenticated Cell Cultures (Shanghai, China), were grown in DMEM (Gibco, Carlsbad, CA, United States) supplemented with 10% fetal bovine serum (Gibco, Carlsbad, CA, United States) and 1% penicillin–streptomycin at 37 °C in a 5% CO_2_ humidified atmosphere incubator. The specific small interfering RNAs targeting ATP6V1C2 (siATP6V1C2) and non-specific control siRNA normal control (siNC) were synthesized by RiboBio (Guangzhou, China), and the sequences are shown as follows: siATP6V1C2-1 CCA​GAT​TGC​TGT​CTG​ATA​A, siATP6V1C2-2 GGA​TGA​AGT​AGC​CGC​TAC​A, and siATP6V1C2-3 GAC​TGC​AAC​TCA​ATA​ACC​A. siATP6V1C2 and siNC were transfected into SW480 and HCT116 cells using Lipofectamine RNAi Max (Thermo Fisher, Waltham, MA, United States) according to the manufacturer’s instructions.

### Cell Proliferation Assays

SW480/HCT116 cells transfected with siATP6V1C2 or siNC were seeded into a 96-well plate, continuously cultured for 120 h, and the number of living cells was measured every 24 h with CCK8 kit (Biosharp, Hefei, Anhui, China). The optical density at 450 nm of each well was quantified using a microplate reader (Allsheng, Hangzou, China).

### Colony Formation Assay

The indicated number of cells were seeded for transfection. When the colonies are clearly visible even without looking under the microscope, colonies were stained with crystal violet and photographed.

### Quantitative Real-Time Polymerase Chain Reaction (qRT-PCR)

Total RNA was extracted from cells using TRIzol reagent, and equal amounts of RNA were used for qRT-PCR analysis (SYBR®Green I, Accurate Biotechnology, Changsha, Hunan) according to the manufacturer’s instructions. GAPDH was used as a loading control. Primers are listed in Supplementary Table S1. The mRNA expression was quantitated using the 2-(^△Ct^ sample–^△Ct^ control) method.

### Western Blot

Proteins from cell lysates were separated by SDS-PAGE and transferred to a PVDF membrane, which was incubated with primary ATP6V1C2 (catalog number: 16274-1-AP) or GAPDH (catalog number: 10494-1-AP) polyclonal antibody (Proteintech, Rosemont, IL, United States), followed by HRP-conjugated secondary antibody. ECL reagent was applied for protein detection. Relative ATP6V1C2 expression was normalized to GAPDH expression at the protein level.

### Immunohistochemistry Staining

The protein expression of ATP6V1C2 in COAD was investigated. Archived formalin-fixed, paraffin-embedded (FFPE) COAD tissue sections were assessed by immunohistochemistry (IHC) staining using ATP6V1C2 polyclonal antibody (Proteintech, Rosemont, IL, United States). Staining was visualized using the DAB Kit (catalog number: GK500710, Gentech, Shanghai, China). Slides were lightly counterstained with hematoxylin. The staining status of ATP6V1C2 was observed under a microscope.

### Statistics Analysis

Differences between the two groups were assessed by a two-tailed, unpaired *t*-test or Student’s *t*-tests. For overall survival (OS) analyses, Kaplan–Meier curves were compared by using the log-rank test. Multivariate analysis with the Cox proportional hazards model was applied. The FDR was used to correct *p*-value. *p*-values or FDR <0.05 were considered to be significant. In this study, *p* < 0.05, <0.01, and <0.001 were marked by one asterisk, two asterisks, or three asterisks, respectively. All statistical analyses were performed in R v4.0.3.

## Results

### ATP6V1C2 Overexpressed in COAD

**FIGURE 1:**
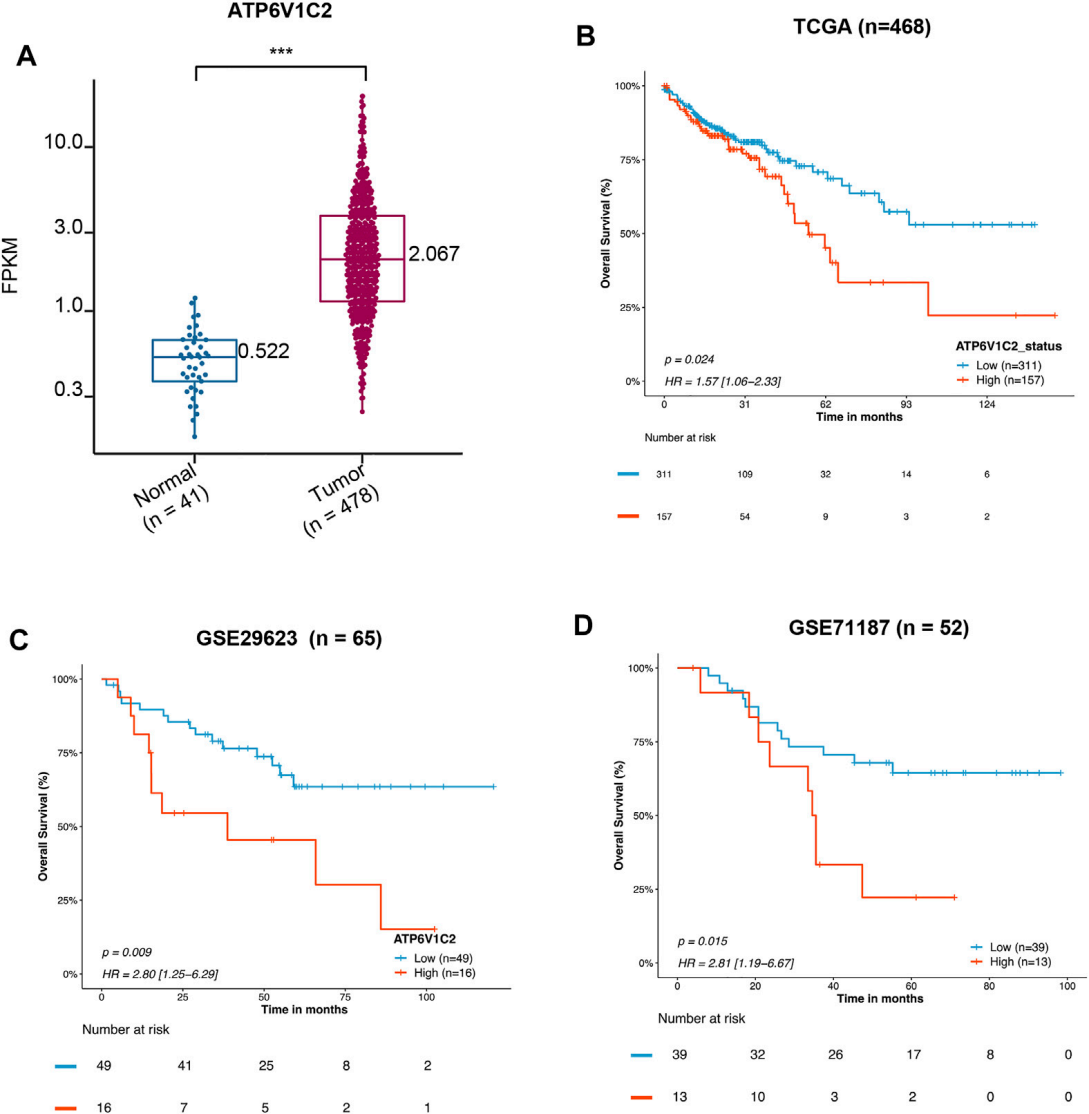
Prognostic value of mRNA expression of ATP6V1C2 in COAD patients. **(A)** The difference in ATP6V1C2 expression level between COAD and adjacent non-cancerous tissues based on TCGA COAD dataset; **(B)** Kaplan–Meier survival curves of overall survival (OS) based on the ATP6V1C2-high and -low groups expression in TCGA-COAD dataset; **(C)** Survival curves of OS based on the ATP6V1C2-high and -low groups expression in GSE29523 dataset; **(D)** Survival curves of OS based on the ATP6V1C2-high and -low groups expression in GSE71187 dataset. COAD: colon adenocarcinoma; TCGA: The Cancer Genome Atlas. *** indicates *p* < 0.001, statistical analysis was performed using two-tailed Student’s *t*-tests.

A total of 41 NC and 478 tumor samples from 468 COAD patients were included in TCGA COAD dataset. The clinicopathological characteristics of patients are summarized in Table 1. We found that ATP6V1C2 at the RNA level was significantly overexpressed in COAD compared with NC with a threshold of log_2_FC > 1 (Figure 1A). Its expression at the protein level was subsequently explored in three archived COAD tissues. ATP6V1C2 was highly expressed in COAD tissues, distributed both in the cytoplasm and on the membrane (Supplementary Figure S1A). The pattern of ATP6V1C2 expression was also retrieved from The Human Protein Atlas (HPA, https://www.proteinatlas.org/). Consistent with our work, ATP6V1C2 staining was observed in the cytoplasm and on the membrane in three COAD patients (Supplementary Figure S1B).

**TABLE 1:** Clinicopathological characteristics of COAD patients.

Characteristics	Overall (*n* = 468)	ATP6V1C2-high (*n* = 157)	ATP6V1C2-low (*n* = 311)	*p*
Age, years				0.106 (fisher.test)
<65	175 (37.39%)	67 (42.68%)	108 (34.73%)	
≥65	293 (62.61%)	90 (57.32%)	203 (65.27%)	
Gender				0.769 (fisher.test)
Female	223 (47.65%)	73 (46.50%)	150 (48.23%)	
Male	245 (52.35%)	84 (53.50%)	161 (51.77%)	
Stage				0.163 (fisher.test)
Stage I	77 (16.45%)	20 (12.74%)	57 (18.33%)	
Stage II	183 (39.10%)	55 (35.03%)	128 (41.16%)	
Stage III	132 (28.21%)	51 (32.48%)	81 (26.05%)	
Stage IV	65 (13.89%)	27 (17.20%)	38 (12.22%)	
Unknown	11 (2.35%)	4 (2.55%)	7 (2.25%)	

COAD, colon adenocarcinoma.

### ATP6V1C2 as an Independent Factor Associated With OS in COAD

**FIGURE 2:**
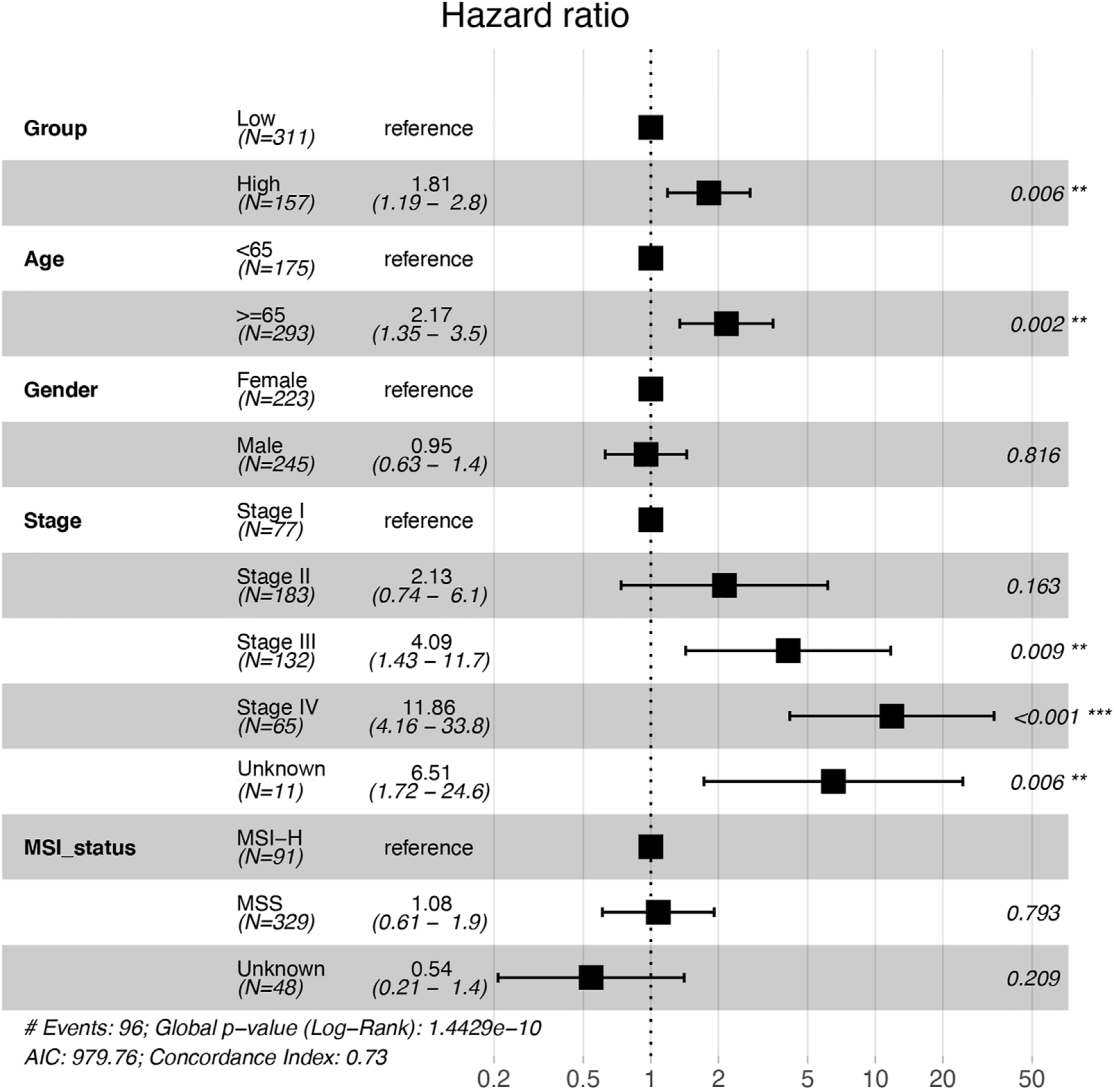
Multivariate analysis with the Cox proportional hazards model on the independent factors associated with overall survival in COAD patients. *, **, and *** indicate *p* < 0.05, *p* < 0.01, and *p* < 0.001, respectively. COAD: colon adenocarcinoma; MSI-H: microsatellite instability-high; MSS: microsatellite stability.

High expression of ATP6V1C2 (cut-off: the FPKM of ATP6V1C2 corresponding to the top one-third of COAD cases; FPKM ≥3.04 [*n* = 157]) was obviously related to a poor OS (*p* = 0.024, 55.37 months vs. not reached [NR], Figure 1B) based on TCGA COAD dataset. In addition, the data from GSE29623 including 65 COAD patients (*p* = 0.009, 38.72 months vs. NR, Figure 1C) and GSE71187 including 52 COAD cases (*p* = 0.015, 35.04 months vs. NR, Figure 1D) further displayed that high expression of ATP6V1C2 [expression ≥6.65 in GSE29623 (*n* = 16) and expression ≥1.24 in GSE71187 (*n* = 13)] predicted a poor OS in COAD. Microsatellite instability-high (MSI-H) tumors represent a subset of COAD tumors, which might be targeted for immune checkpoint inhibitors. Compared with NC, ATP6V1C2 was overexpressed both in microsatellite stability (MSS) and MSI-H tumors (Supplementary Figure S2A), while it was associated with OS in MSS tumors (*p* = 0.031, 49.77 months in ATP6V1C2-high group vs. NR in ATP6V1C2-low group, Supplementary Figure S2B) rather than MSI-H tumors (*p* = 0.405, NR in ATP6V1C2-high group vs. 71.13 months in ATP6V1C2-low group, Supplementary Figure S2C) according to the TCGA COAD dataset. Next, whether high expression of ATP6V1C2 is an independent factor associated with OS was evaluated. Multivariate analysis with the Cox proportional hazards model revealed that ATP6V1C2 expression was an independent factor associated with OS in COAD (*p* = 0.006, Figure 2).

### Interaction Networks Between ATP6V1C2 and Its Interactive Genes

**FIGURE 3:**
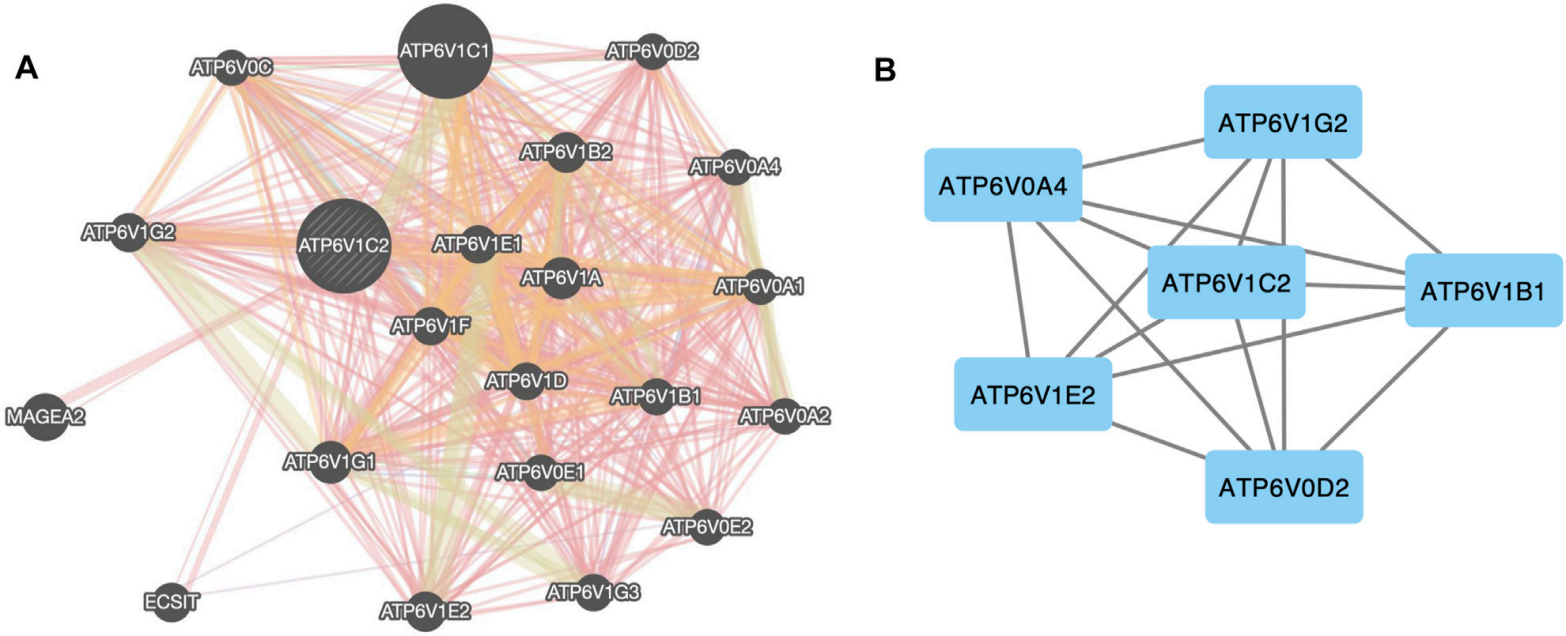
Protein–protein network of ATP6V1C2. **(A)** Protein–protein network between ATP6V1C2 and top 20 co-expressed genes predicted from GeneMANIA database; **(B)** Protein–protein network between ATP6V1C2 and DEGs in COAD among these top 20 genes. DEG: differentially expressed gene; COAD: colon adenocarcinoma.

We analyzed the interactive networks of ATP6V1C2 with its interactive genes using the GeneMANIA (Figure 3). In GeneMANIA database, top 20 ATP6V1C2-related genes were enriched (Figure 3A), and most of them (18 ATPs gene) were ATP family members (except ECSIT and MAGEA2). Among these top 20 genes, ATP6V0A4 (log_2_FC = 4.52, *p* = 1.89e-18), ATP6V1B1 (log_2_FC = 2.15, *p* = 6.08e-13), ATP6V1E2 (log_2_FC = 1.17, *p* = 2.73e-33), and ATP6V1C2 (log_2_FC = 2.37, *p* = 1.76e-36) were up-regulated. However, ATP6V0D2 (log_2_FC = -2.25, *p* = 3.85e-27) and ATP6V1G2 (log_2_FC = -2.46, *p* = 2.15e-51) were down-regulated. These six genes were differentially expressed between normal and tumor samples in the TCGA COAD dataset. Further analysis with STRING database demonstrated that these six genes interacted with each other (Figure 3B).

### Association of ATP6V1C2 Expression With EMT Process

**FIGURE 4:**
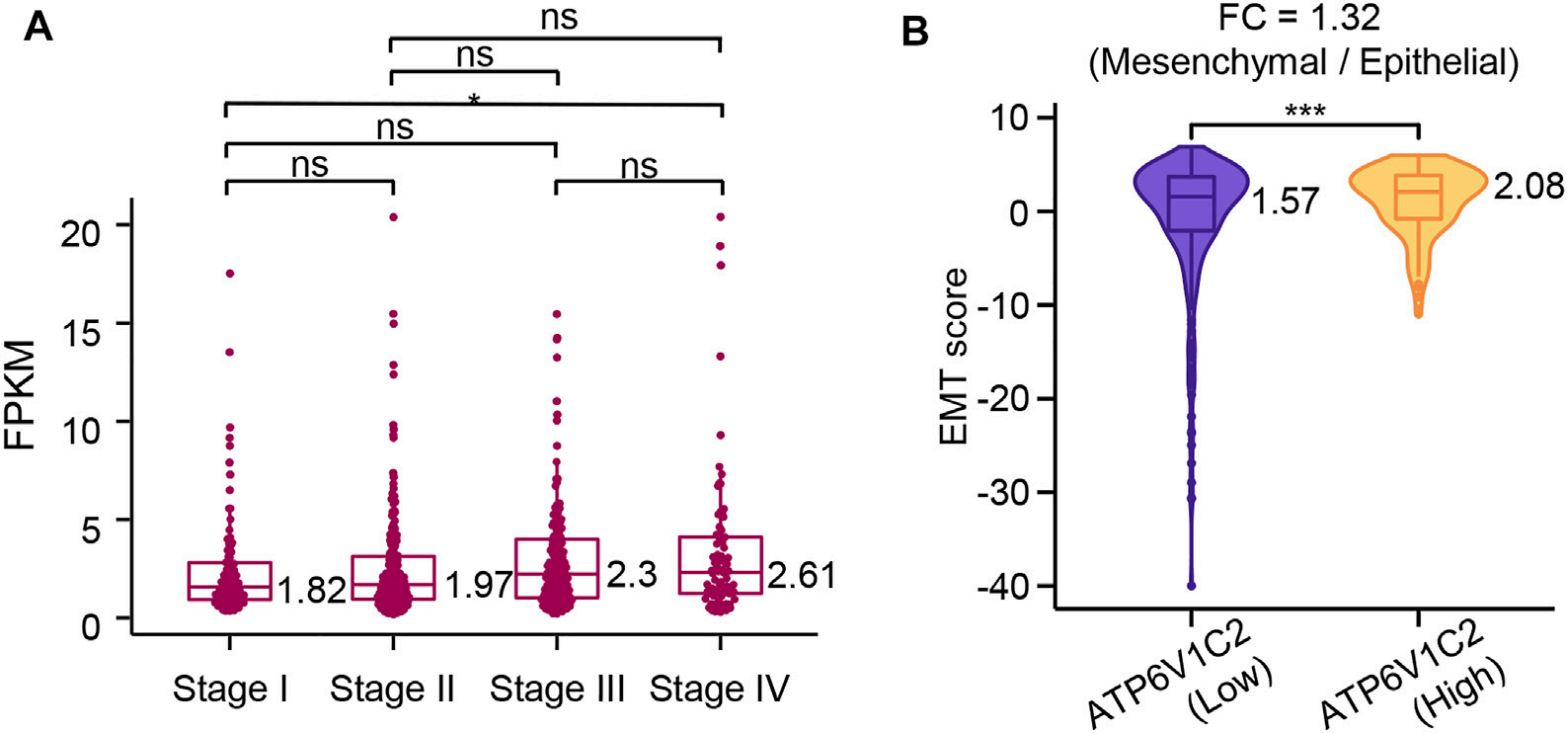
The association of ATP6V1C2 expression with EMT process. **(A)** The difference in ATP6V1C2 expression level among patients with stage I, stage II, stage III, and stage IV; **(B)** The difference in EMT score between ATP6V1C2-high and -low groups. ns indicates *p* > 0.05. * indicates *p* < 0.05. *** indicates *p* < 0.001. EMT, epithelial–mesenchymal transition. FPKM: Fragments per kilobase of exon model per million mapped fragments; FC: fold change; EMT: epithelial–mesenchymal transition.

ATP6V1C2 showed a significantly higher level in metastatic COAD (stage IV) than that in early-stage COAD (stage I) (*p* = 0.046, Figure 4A) according to the TCGA COAD dataset, which indicated that ATP6V1C2 might be involved in COAD metastasis. EMT is a cellular process implicated in cancer metastasis. The EMT status of cancer cells can prove to be a critical estimate of patient prognosis. In the present work, EMT score was calculated based on a 76-gene EMT signature as previously described. We found that ATP6V1C2-high group displayed a significantly higher EMT score with a FC of 1.32 compared with ATP6V1C2-low group (2.08 vs. 1.57, *p* = 0.0003, Figure 4B). These data suggest that ATP6V1C2 might be involved in EMT of COAD.

### Association of ATP6V1C2 Expression With TME Contexture

**FIGURE 5:**
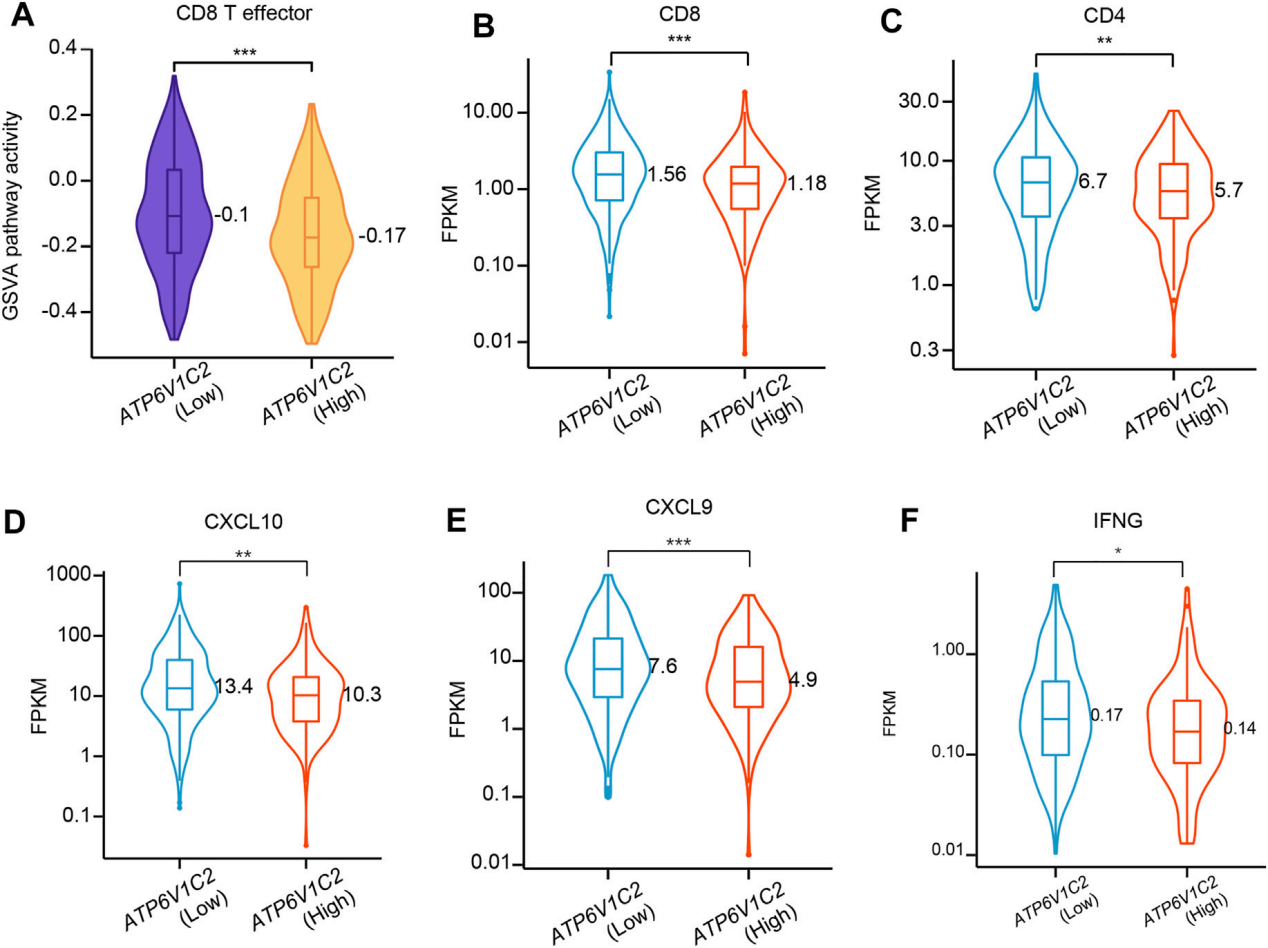
The difference in CD8 T effector pathway activity between ATP6V1C2-high and -low groups. **(A)** CD8 T effector pathway activity; **(B)** CD8 expression; **(C)** CD4 expression; **(D)** CXCL10 expression; **(E)** CXCL9 expression; **(F)** IFNG expression. *, **, and *** indicate *p* < 0.05, *p* < 0.01, and *p* < 0.001, respectively. FPKM: Fragments per kilobase of exon model per million mapped fragments.

Tumor microenvironment (TME) presents a coordinated network of interface cell types mainly including immune cells, endothelial cells, and fibroblasts through the extracellular matrix, cytokines, chemokines, and growth factors to influence the development and progression of cancer. In this study, whether ATP6V1C2 expression level is related to TME contexture was investigated. In this study, we found that ATP6V1C2-high group had significantly decreased GSVA pathway activity in CD8 T effector (Figure 5A). The expression levels of CD8 (*p* = 0.0006, Figure 5B), CD4 (*p* = 0.009, Figure 5C), CXCL10 (*p* = 0.001, Figure 5D), CXCL9 (*p* = 0.0005, Figure 5E), and IFNG (*p* = 0.014, Figure 5F) in ATP6V1C2-high group were also significantly lower than those in ATP6V1C2-low group.

### Potential Molecular Mechanisms of ATP6V1C2 Implicated in COAD Tumorigenesis

**FIGURE 6:**
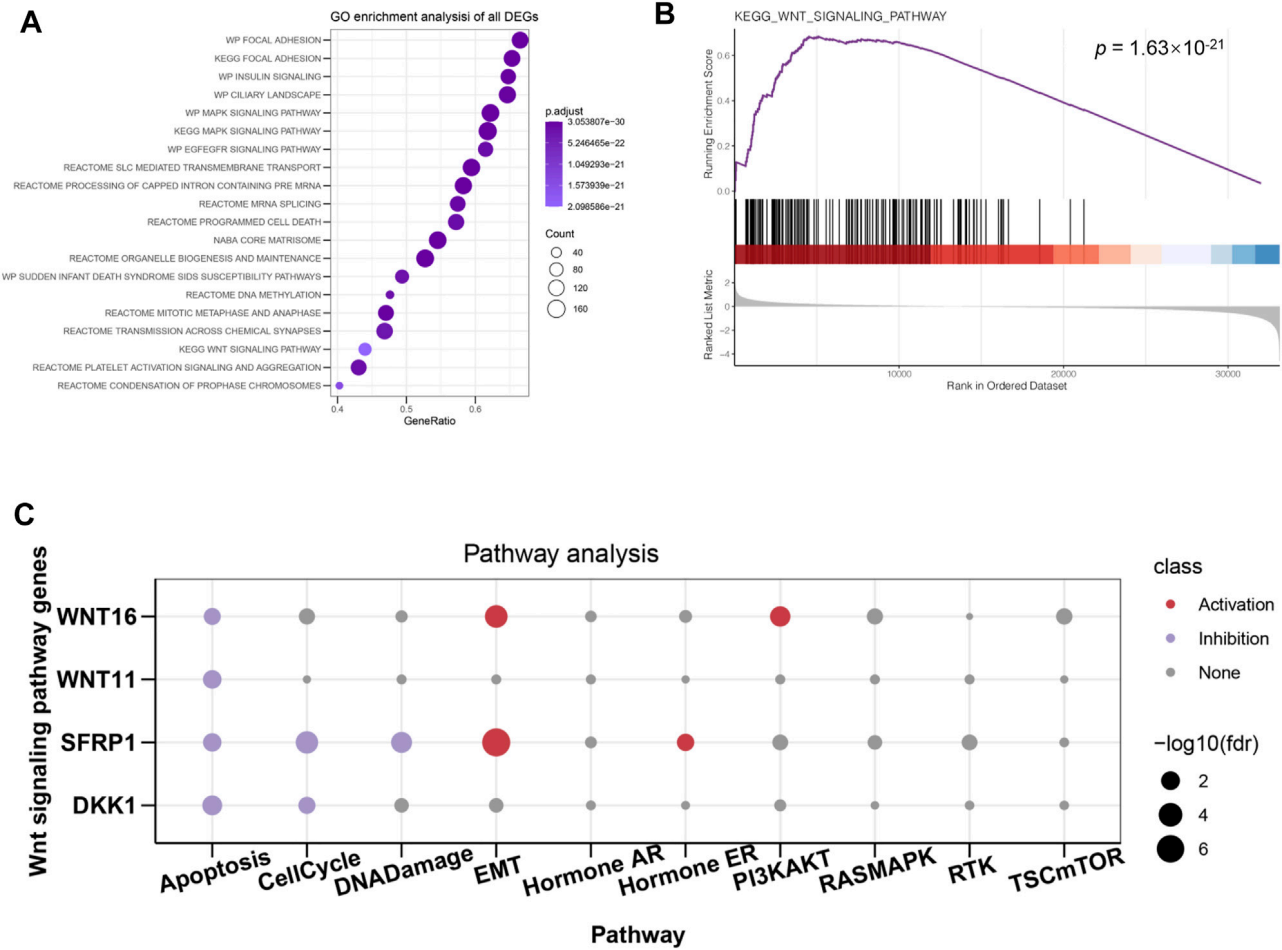
DEGs between ATP6V1C2-high and -low group and the potential roles of identified DEGs. **(A)** GO and KEGG enrichment of DEGs; **(B)** Wnt signaling pathway enriched from identified DEGs; **(C)** The associations between Wnt hub genes and cellular processes/signaling pathways implicated in tumor progression. DEGs: differentially expressed genes; GO: Gene Ontology; KEGG: Kyoto Encyclopedia of Genes and Genomes; EMT: epithelial–mesenchymal transition.

In order to further investigate the molecular mechanisms of ATP6V1C2 involved in COAD, DEGs between ATP6V1C2-high and -low groups were identified in the TCGA COAD cohort. A total of 170 up- and 708 down-regulated DEGs were identified in ATP6V1C2-high group, which were significantly enriched in Wnt (*p* = 1.498e-23, Figure 6A) signaling pathway. GSEA analysis indicated that DEGs related to high expression of ATP6V1C2 were enriched in Wnt pathway module (*p* = 1.63e-21 Figure 6B). A total of four DEGs implicated in Wnt pathway were identified in ATP6V1C2-high group vs. ATP6V1C2-low group, including down-regulated DDK1, down-regulated SFPR1, up-regulated WNT11, and up-regulated WNT16. Next, the crosstalk between these four DEGs and EMT process was explored based on GSCA. We found that SFRP1 and WNT16 displayed high activity of EMT (Figure 6C). Taken together, Wnt signaling pathway might play key roles in COAD tumorigenesis by regulating EMT process.

### Exploration of Biological Roles of ATP6V1C2 in COAD Tumorigenesis Through *in vitro* Studies

**FIGURE 7:**
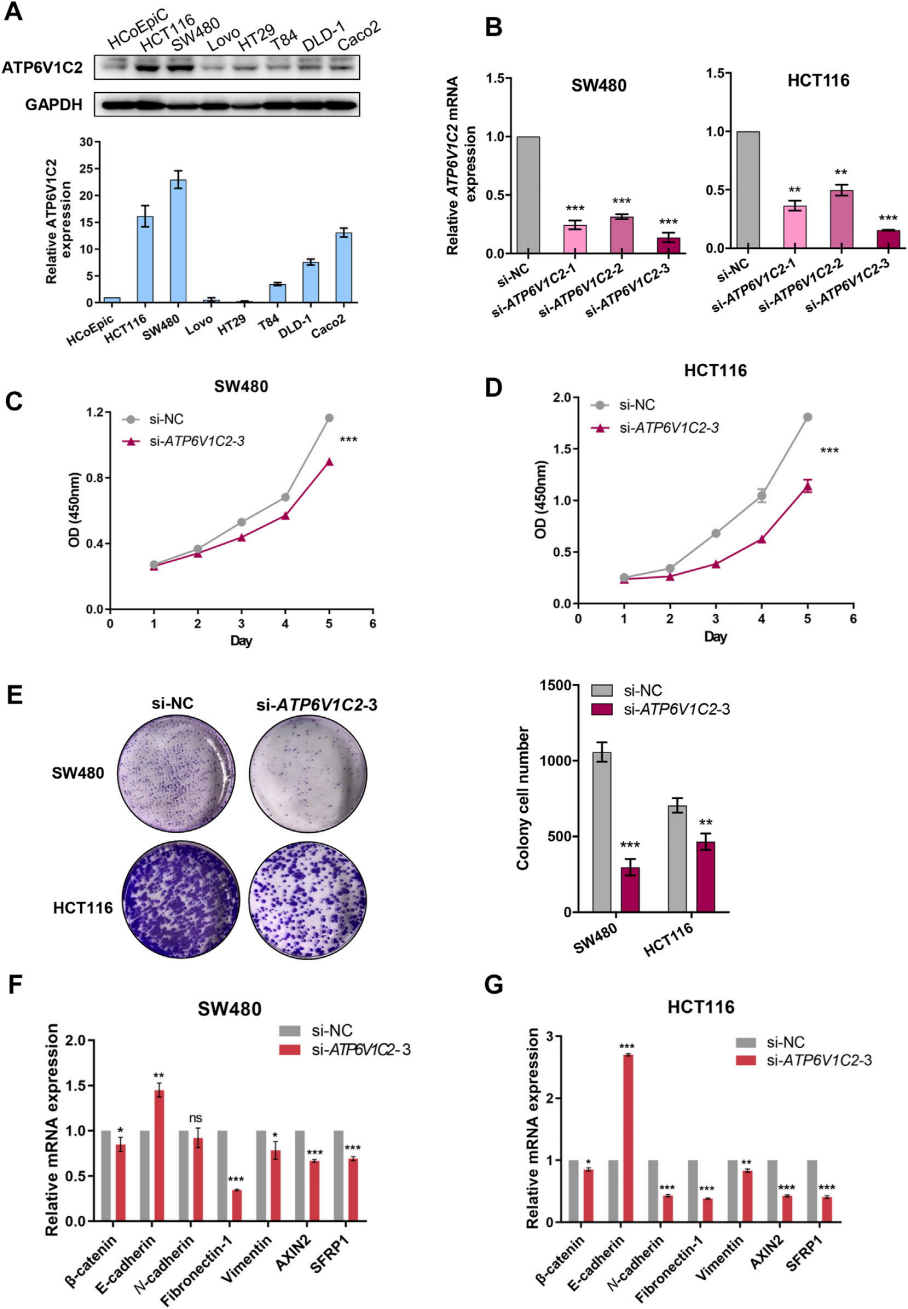
*In vitro* studies on ATP6V1C2 in COAD tumorigenesis. **(A)** The protein expression level of ATP6V1C2 in human colonic epithelial cells (HCoEpiC) and COAD cells; **(B)** The mRNA expression level of ATP6V1C2 in SW480/HCT116 cells transfected with siATP6V1C2 and siNC; **(C)** Proliferation curves assessed by CCK8 assay during 120 h for ATP6V1C2 knockdown cell model of SW480; **(D)** Proliferation curves assessed by CCK8 assay during 120 h for ATP6V1C2 knockdown cell model of HCT116; **(E)** The cloning ability for ATP6V1C2 knockdown cell models of SW480 and HCT116; The expression level of Wnt pathway-related and EMT-related genes in SW480 **(F)** and HCT116 cells **(G)** with siATP6V1C2. COAD: colon adenocarcinoma. *, **, and *** indicate *p* < 0.05, *p* < 0.01, and *p* < 0.001, respectively. OD: optical density; NC: normal control.

In this work, ATP6V1C2 expression status was investigated in several cell lines, including human colonic epithelial cells (HCoEpiC) and COAD cells (HCT116, SW480, LOVO, T84, DLD-1, and Caco2). We found that ATP6V1C2 had a high protein expression level both in HCT116 and SW480 (Figure 7A). In order to investigate the potential biological roles in COAD, *in vitro* studies including cell proliferation assay and colony formation analysis were performed to evaluate the effects of ATP6V1C2 knockdown on COAD cell proliferation. Compared with normal controls, COAD cell lines SW480 and HCT116 presented significantly decreased ATP6V1C2 expression at the mRNA level 48 h post-transfection with siATP6V1C2 (Figure 7B). We found the significantly decreased cell proliferation ability of COAD cell lines SW480 (Figure 7C, *p* < 0.0001) and HCT116 (Figure 7D, *p* = 0.0004) with ATP6V1C2 knockdown. Furthermore, cloning ability of SW480 (*p* < 0.0001) and HCT116 cells was also inhibited (*p* = 0.005) (Figure 7E). The expression levels of the Wnt pathway-related and EMT-related genes were explored in COAD cell lines with knockdown of ATP6V1C2. We found that β-catenin as an intracellular Wnt effector, AXIN2 as a Wnt/β-catenin target gene, EMT-related genes (including fibronectin-1 [FN1] and vimentin [VIM]) were significantly decreased, and another EMT-related gene E-cadherin (CDH1) was dramatically increased in both SW480 (Figure 7F) and HCT116 cells (Figure 7G) with silencing ATP6V1C2. These findings suggest that ATP6V1C2 might enhance cell proliferation and growth via β-catenin-mediated EMT in COAD.

## Discussion

Despite recent advances in understanding the biomedical and molecular mechanisms of COAD, the prognosis of COAD patients remains unfavorable. Further understating of the molecular changes in COAD may lead to new and effective therapies toward prevention of COAD. To the best of our knowledge, this is the first study to demonstrate the associations of ATP6V1s family members with survival outcomes in COAD. Moreover, the potential molecular mechanisms of ATP6V1C2 implicated in COAD tumorigenesis were also investigated based on public datasets and *in vitro* studies.

ATP6V1s family members as the components of V-ATPase have been documented to participate in the development of various tumors, such as ATP6V1A implicated in gastric cancer (Wang et al., 2017), ATP6V1C1 implicated in breast cancer (Feng et al., 2013; Cai et al., 2014), esophageal squamous cell carcinoma (Yao et al., 2021), and oral squamous cell carcinoma (Pérez-Sayáns et al., 2010), ATP6V1G1 implicated in glioblastoma (Di Cristofori et al., 2015), and ATP6V1G3 implicated in renal cell carcinoma (Shinmura et al., 2015). ATP6V1C2 encodes ATPase H+ transporting V1 subunit C2, which has been documented to be involved in renal clear cell carcinoma (RCC) other than breast cancer (BC) (McConnell et al., 2017; Li et al., 2020). Its biological or prognostic roles in COAD remain elusive. Results from our study showed that ATP6V1C2 expression was significantly up-regulated in tissues of patients with COAD and correlated with shorter overall survival independent of clinical variables. The expression status of ATP6V1C2 in COAD in this study differed from that in RCC and BC reported in previous studies (McConnell et al., 2017; Li et al., 2020). Li and colleagues (Li et al., 2020) have reported that ATP6V1C2 is significantly down-regulated in RCC tissues and high expression of ATP6V1C2 predicts shorter overall survival in RCC patients from TCGA. For BC, ATP6V1C2 is only overexpressed or gnomically amplified in 7% of cases, and its overexpression or genomic amplification did not significantly correspond to effects on patients’ survival (McConnell et al., 2017). The previous and our studies suggest that ATP6V1C2 displays distinct expression status, and prognostic role among different cancers and the molecular mechanisms of ATP6V1C2 related to COAD, RCC, and BC are different. Our study reveals that high expression of ATP6V1C2 predicts unfavorable overall survival outcomes in COAD patients. MSI-H tumors account for 10%–15% of COAD cases and have improved efficacy with immunotherapy. In this study, high expression of ATP6V1C2 showed a longer OS in MSS tumors rather than MSI-H tumors. Whether ATP6V1C2 could predict the prognosis of MSI-H patients with immunotherapy should be further investigated.

EMT is a process during which cells lose their epithelial characteristics, for instance, cell–cell contact, and gain mesenchymal properties, such as increased motility (Pastushenko and Blanpain, 2019; Babaei et al., 2021). In CRC, EMT contributes to tumor metastasis and increased resistance to chemotherapy and immunotherapy (Zhang et al., 2021). In this study, EMT score was calculated based on the 76GS method as previously reported, which indicates that high EMT score (76GS score) suggests more epithelial features. Our results revealed that ATP6V1C2-high group had a significantly higher EMT score, in addition, we also found that metastatic COAD (stage IV) patients had dramatically overexpressed ATP6V1C2 compared with early-stage (stage I) patients. These findings suggest that ATP6V1C2-high group is more prone to display EMT phenotype compared with ATP6V1C2-low group, resulting in greater metastatic potential.

Tumors evolve in close interaction with their microenvironment, which encompasses a continual tension between the developing tumor and the host immune system (Angell et al., 2020). The TME is comprised of a variety of cellular entities (including cells of immune system, also knowns as tumor-infiltrating lymphocytes) and several soluble factors within the tumor (including cytokines and chemokines) (Angell et al., 2020). Previous studies have documented that TME impacts the tumor progression and patient survival (Galon et al., 2012). In this study, we found that ATP6V1C2-high group had lower CD8 T effector pathway activity and decreased expression of CD4, CD8, CXCL9, and CXCL10. These findings suggest that ATP6V1C2 might hinder immune system in defense against tumor cells, resulting in a poor prognosis of COAD patients with high level of ATP6V1C2.

**FIGURE 8:**
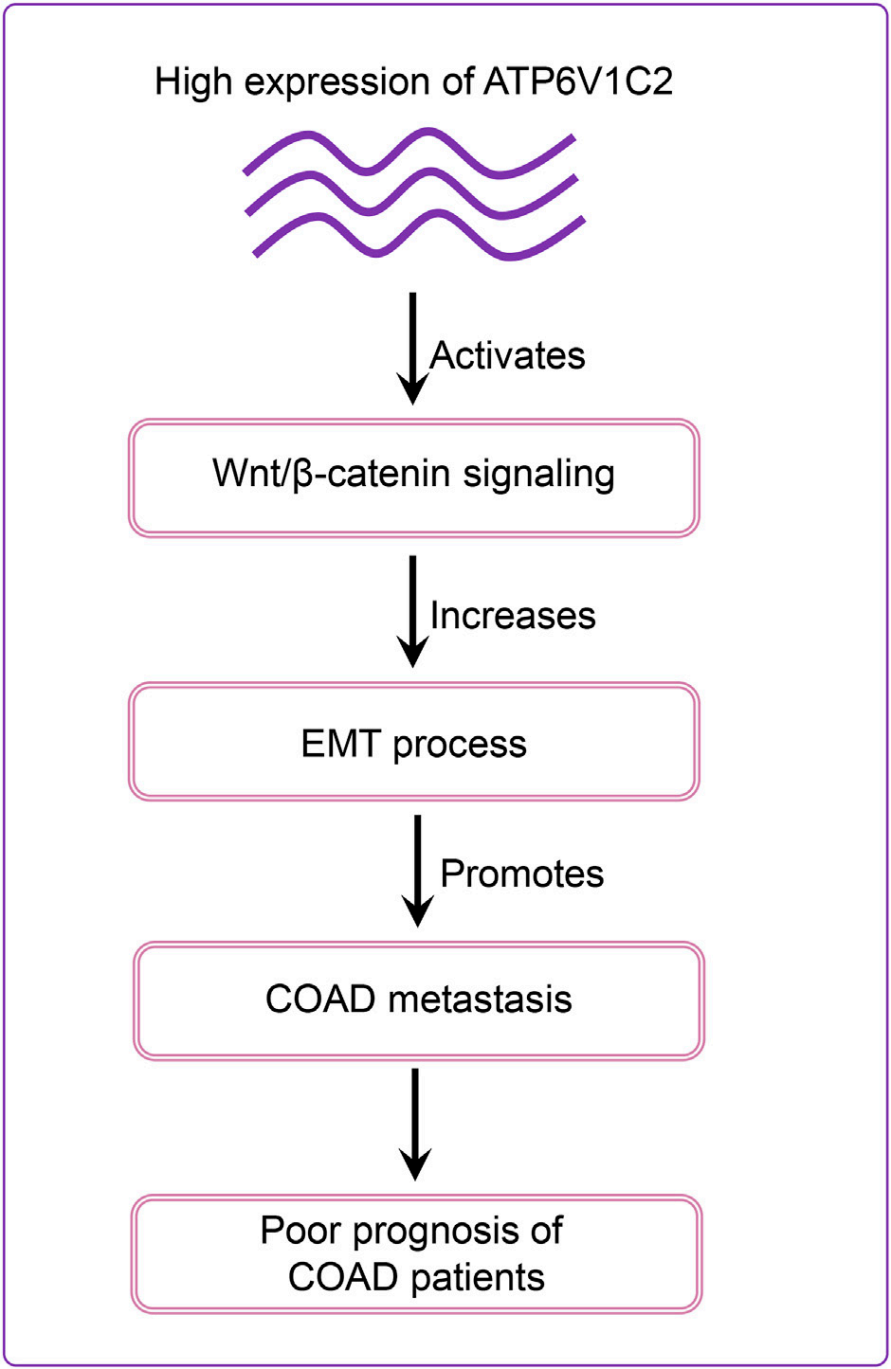
Schematic diagram of ATP6V1C2 expression on the prognosis of COAD patients. ATP6V1C2 overexpression might promote EMT cellular process by activating Wnt signaling pathway, resulting in cancer metastasis and unfavorable prognosis for COAD patients. COAD: colon adenocarcinoma; EMT: epithelial–mesenchymal transition.

Our results revealed that DEGs between ATP6V1C2-high and -low groups were significantly enriched in Wnt signaling pathway. In addition, several Wnt signaling pathways-related genes were significantly correlated with EMT activation, which was consistent with previous studies indicating that Wnt pathway promotes malignant progression by regulating the EMT in colon cancer (Mosa et al., 2020; Yi et al., 2020). *In vitro* studies revealed that ATP6V1C2 knockdown resulted in aberrant expression of Wnt pathway-related and EMT-related genes. Based on these findings, we proposed a model to speculate the roles of ATP6V1C2 in promoting COAD progression and metastasis (Figure 8). In detail, ATP6V1C2 overexpression could promote EMT cellular process by activating Wnt signaling pathway, then resulting in COAD metastasis and poor prognosis for COAD patients.

There were some limitations in the study. First, *in vitro* studies were performed to preliminarily evaluate the biological roles of ATP6V1C2 in COAD tumorigenesis. *In vivo* studies are warranted to investigate the molecular mechanisms of ATP6V1C2 in COAD tumorigenesis. Second, the prognostic value of ATP6V1C2 was investigated in TCGA and GEO datasets in this study. A large, prospective, multi-center cohort study is needed to explore its prognostic value in COAD patients. Third, different cut-offs of ATP6V1C2 expression in predicting OS were used, such as FPKM ≥3.04 in TCGA-COAD, expression≥6.65 in GSE29623, and expression≥1.24 in GSE71187. Further studies are needed to explore the cut-offs of ATPV1C2 expression detected using different platforms with high performance, such as qRT-PCR and RNA sequencing, in predicting OS of COAD patients. Whether ATP6V1C2 expression is a feasible tool in predicting OS of COAD patients and whether ATP6V1C2 expression detection enables clinical physicians to stratify COAD patients for management are needed to be explored.

This is the first study to report the biological roles of ATP6V1C2 in COAD. Based on our bioinformatics analyses and *in vitro* experiments, we reported the potential molecular mechanisms of ATP6V1C2 implicated in COAD tumorigenesis. OS analysis revealed that high expression of ATP6V1C2 predicted poor prognosis in COAD. ATP6V1C2 was identified as an independent factor associated with OS in COAD. *In vitro* experiment revealed that ATP6V1C2 knockdown resulted in aberrant expression of Wnt- and EMT-related genes and inhibited COAD cell proliferation and growth. Furthermore, this study indicates that ATP6V1C2 might be a novel therapeutical target in COAD.

## Data Availability

The original contributions presented in the study are included in the article/Supplementary Material, and further inquiries can be directed to the corresponding authors.
