# Supplementary figures and images for: High Expression of ATP6V1C2 Predicts Unfavorable Overall Survival in Patients With Colon Adenocarcinoma
Source: Front Genet. 2022 Sep 21;13:930876. doi: 10.3389/fgene.2022.930876 (PMC9532742; doi:10.3389/fgene.2022.930876)

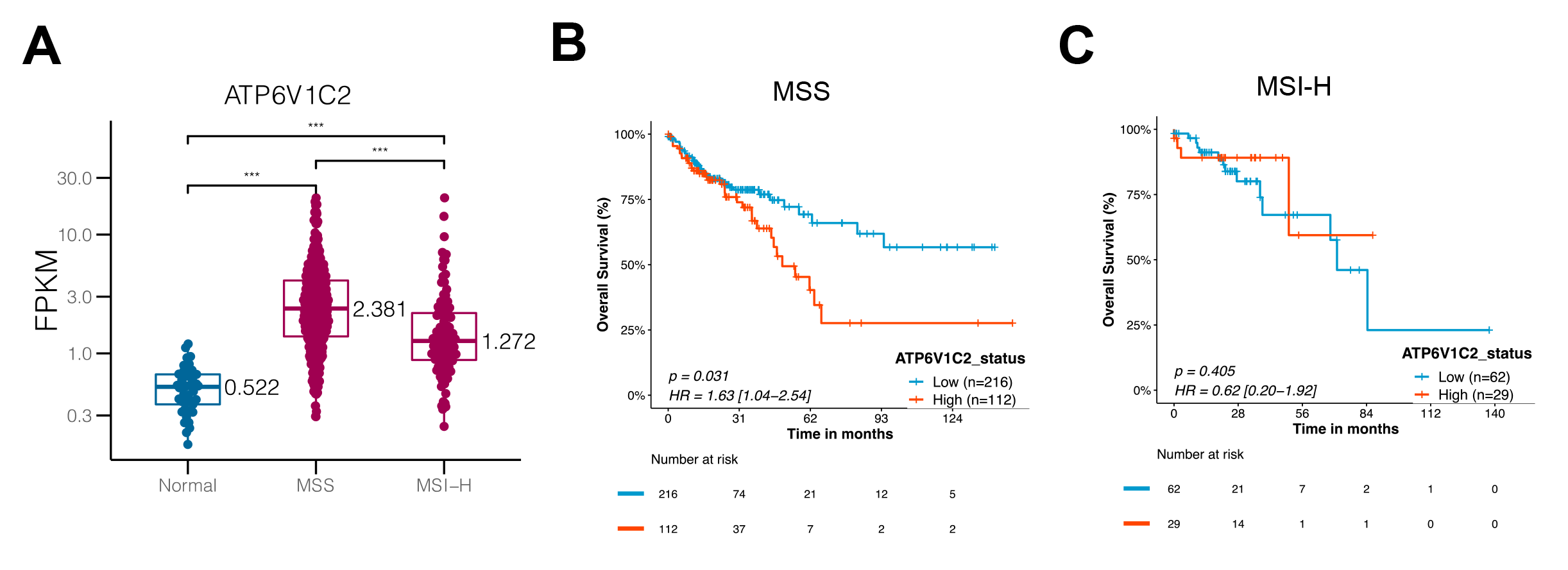

Supplement: Supplementary file 1 [file Image2.TIF]

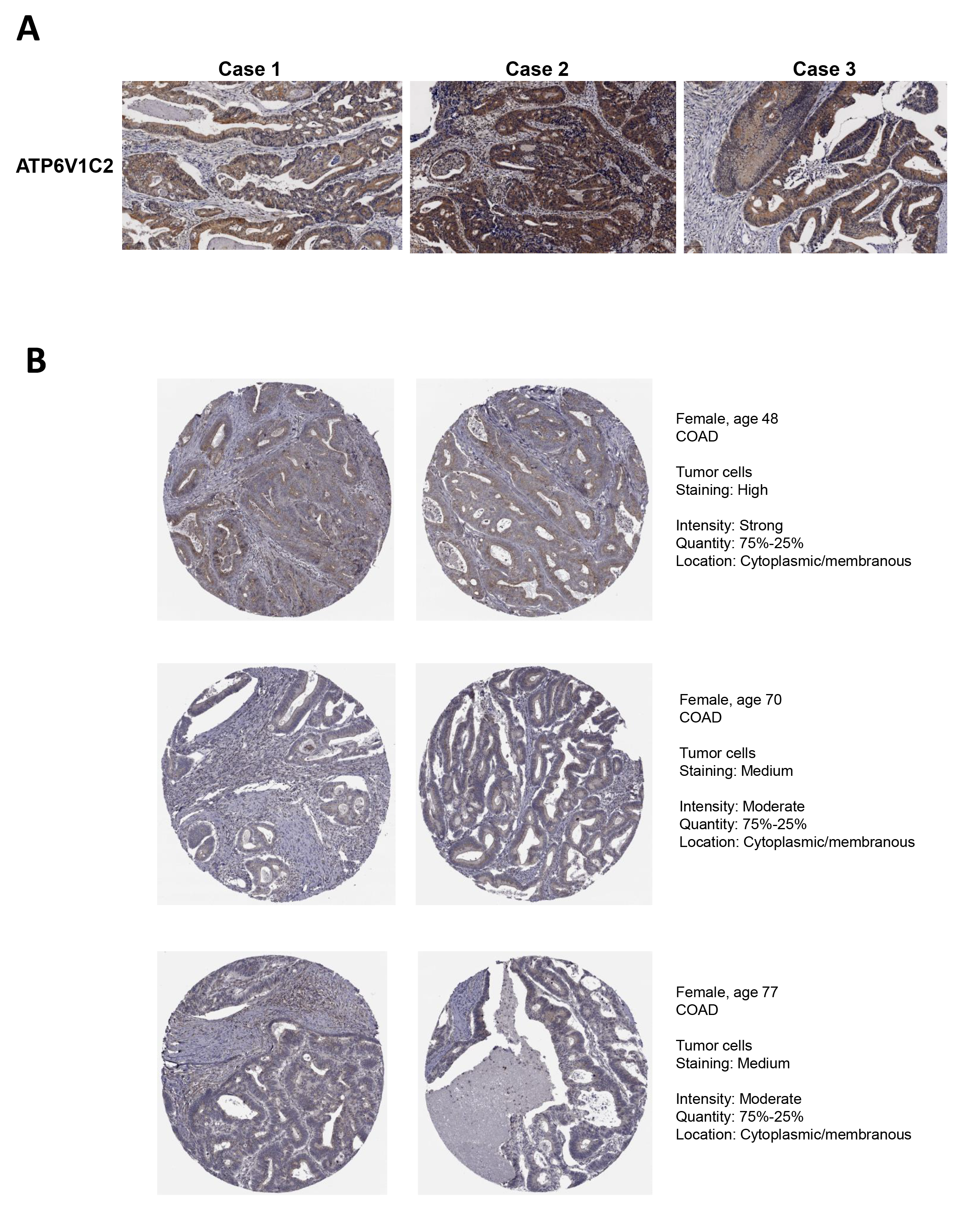

Supplement: Supplementary file 2 [file Image1.TIF]
