# Supplementary material for: High Expression of ATP6V1C2 Predicts Unfavorable Overall Survival in Patients With Colon Adenocarcinoma
Source: Front Genet. 2022 Sep 21;13:930876. doi: 10.3389/fgene.2022.930876 (PMC9532742; doi:10.3389/fgene.2022.930876)
Supplement: Supplementary file 3 [file Table2.DOCX]

**Table S1 list of primers for qRT-PCR**

| **Gene** | **Primer** |
| --- | --- |
| β-Catenin | F: CATCTACACAGTTTGATGCTGCT |
|  | R: GCAGTTTTGTCAGTTCAGGGA |
| E-cadherin | F: CGAGAGCTACACGTTCACGG |
|  | R: GGGTGTCGAGGGAAAAATAGG |
| N-cadherin | F: TCAGGCGTCTGTAGAGGCTT |
|  | R: ATGCACATCCTTCGATAAGACTG |
| Fibronectin-1 | F: CGGTGGCTGTCAGTCAAAG |
|  | R: AAACCTCGGCTTCCTCCATAA |
| Vimentin | F: CCCTCACCTGTGAAGTGGAT |
|  | R: GCTTCAACGGCAAAGTTCTC |
| AXIN2 | F: CAACACCAGGCGGAACGAA |
|  | R: GCCCAATAAGGAGTGTAAGGACT |
| ATP6V1C2 | F: TTTCTGCCCCTGGCGATAAG |
|  | R: AAAGGTGTCGAGTTTCCCCAA |
| GAPDH | F: AGCCACATCGCTCAGACAC |
|  | R: GCCCAATACGACCAAATCC |

qRT-PCR: qualitative real-time polymerase chain reaction; F: forward primer; R: reverse primer.
